# Mouse Thyroid Gland Changes in Aging: Implication of Galectin-3 and Sphingomyelinase

**DOI:** 10.1155/2017/8102170

**Published:** 2017-12-11

**Authors:** Giovanna Traina, Samuela Cataldi, Paola Siccu, Elisabetta Loreti, Ivana Ferri, Angelo Sidoni, Michela Codini, Chiara Gizzi, Marzia Sichetti, Francesco Saverio Ambesi-Impiombato, Tommaso Beccari, Francesco Curcio, Elisabetta Albi

**Affiliations:** ^1^Department of Pharmaceutical Sciences, University of Perugia, Perugia, Italy; ^2^Division of Anatomic Pathology and Histology, Department of Experimental Medicine, School of Medicine and Surgery, University of Perugia, Perugia, Italy; ^3^Department of Medica Area, University of Udine, P.le M. Kolbe 4, 33100 Udine, Italy

## Abstract

Prevalence of thyroid dysfunction and its impact on cognition in older people has been demonstrated, but many points remain unclarified. In order to study the effect of aging on the thyroid gland, we compared the thyroid gland of very old mice with that of younger ones. We have first investigated the changes of thyroid microstructure and the possibility that molecules involved in thyroid function might be associated with structural changes. Results from this study indicate changes in the height of the thyrocytes and in the amplitude of interfollicular spaces, anomalous expression/localization of thyrotropin, thyrotropin receptor, and thyroglobulin aging. Thyrotropin and thyrotropin receptor are upregulated and are distributed inside the colloid while thyroglobulin fills the interfollicular spaces. In an approach aimed at defining the behavior of molecules that change in different physiopathological conditions of thyroid, such as galectin-3 and sphingomyelinase, we then wondered what was their behavior in the thyroid gland in aging. Importantly, in comparison with the thyroid of young animals, we have found a higher expression of galectin-3 and a delocalization of neutral sphingomyelinase in the thyroid of old animals. A possible relationship between galectin-3, neutral sphingomyelinase, and aging has been discussed.

## 1. Introduction

Galectins are a family of proteins with specific domains of 130 amino acids able to bind *β*-galactosides [1]. 15 mammalian galectins are classified into three groups: prototype galectins, tandem galectins, and chimera-type group, of which galectin-3 (Gal-3) is the only member [2]. Gal-3 is widely distributed in a large number of tissues, and at the cellular level, it can be located in the membranes, cytoplasm, and nucleus [3]. Gal-3 is known to modulate many immune reactions [4]. It can be released extracellularly under different inflammatory stimuli like lipopolysaccharide, known to induce upregulation of Gal-3 expression [5]. Moreover, Gal-3 plays a role in leukocyte recruitment to the inflamed microcirculation [6]. Recent evidence shows that Gal-3 plays a role in numerous pathologic conditions such as inflammation [7], metabolic disorders [8], and cancer [9]. In humans, Gal-3 is upregulated in malignant thyroid neoplasms as compared to benign neoplasms and in particular in papillary thyroid carcinoma (PTC), the most prevalent type of malignant tumor of the endocrine system [10]. In rats, the microgravity induces the upregulation of Gal-3 in thyrocytes as well as its release in colloid [11].

Increasing studies demonstrate that neutral sphingomyelinase (nSMase), also known as sphingomyelin phosphodiesterase, an enzyme that uses sphingomyelin (SM) as a substrate to produce ceramide and phosphocholine, may regulate many cell physiopathology processes [12]. Recently, it has been demonstrated that nSMase is upregulated in aging [13]. nSMase is present in the thyrocytes of the thyroid gland, and it is more expressed in the right than in the left lobe [14]. In thyrocytes in culture, the nSMase activity depends on their physiological state by influencing, together with sphingomyelin synthase (SM-synthase), the ceramide/diacylglycerol balance [15]. Thyroid nSMase activity is stimulated during spaceflight and after ionizing and nonionizing ray treatment [16].

At the moment, nothing is known about Gal-3 and nSMase behavior in the thyroid gland during aging and, therefore, the aim of the present study was to investigate this point.

## 2. Methods

### 2.1. Animals

Three CD-1 male mice six weeks old and three CD-1 male mice eighteen months old (Harlan Laboratories Srl, Correzzana D'Adda, Milan, Italy) were used. Mice were kept under a 12-hour light/dark cycle and housed under controlled conditions as reported in Traina et al. [17]. Mice had free access to pelleted food and water. The weight was 26 ± 3 g and 47 ± 2 g for young and old animals, respectively.

### 2.2. Ethical Approval

The experimental protocol was approved by the Ethical Committee for Animal Experimentation at the University of Perugia, Italy. Animal care was in compliance with Italian regulations (Ministerial Declaration 04.03.2014 n°26), as well as with European Economic Community regulations (O.J. of European Commission 2010/63/UE).

### 2.3. Reagents

Anti-TSH receptor (TSHR), anti-nSMase, and fluorescein isothiocyanate- (FITC-) conjugated secondary antibody were obtained from Santa Cruz Biotechnology Inc. (California, USA). TSH, thyroglobulin, and Gal-3 antibodies were from Leica Biosystems (Newcastle Ltd., UK).

### 2.4. Thyroid Tissue Treatment

The thyroid tissue was fixed in 4% neutral phosphate-buffered formaldehyde solution for 24 h, as previously reported [14]. 4 *μ*m thick sections were prepared and mounted on silane-coated glass slides, two for a slide at a distance equal to 140 *μ*m. Between 7 and 24 pairs, sections were sampled excluding the first and the last; sections 7, 13, and 19 were used for hematoxylin-eosin staining; 8, 14, and 20 for TSH detection; 9, 15, and 21 for thyroglobulin detection; 10, 16, and 22 for TSHR detection; 11, 17, and 23 for Gal-3 detection; and 12, 18, and 24 for nSMase detection. Tissue sections were deparaffinized and rehydrated with a series of xylene and ethanol washes.

### 2.5. Morphological Analysis

The sections were stained by the hematoxylin-eosin (Chroma-Gesellschaft, Germany) staining method and investigated by using an inverted microscope, EUROMEX FE 2935 (ED Amhem, The Netherlands), equipped with a CMEX 5000 camera system (40x magnification), as previously reported [14]. The morphometric analysis was performed by using ImageFocus software.

### 2.6. Immunohistochemical Analysis

To remove paraffin from tissue sections before rehydration and immunostaining on the Bond automated system (Leica Biosystems Newcastle Ltd., UK), Bond Dewax solution was used as previously reported [14]. Immunostaining detection was performed by using TSH, thyroglobulin, and Gal-3 antibodies. Bond Polymer Refine Detection was from Leica Biosystems (Newcastle Ltd., UK) [14]. The images were investigated by using microscopy as reported for morphological analysis at 40x magnification.

### 2.7. Immunofluorescence Analysis

After 3 washes with phosphate-buffered saline (PBS), sections were incubated with 2 *μ*g/ml anti-TSHR or anti-nSMase primary antibodies diluted in a 0.5% solution of bovine serum albumin (BSA) in PBS overnight at 4°C. The slides were washed 3 times with PBS and incubated with fluorochrome-conjugated secondary antibodies for 1 hour at room temperature. Then, after 3 washes with PBS, the slides were mounted with glycerol and coverslips. The samples were examined under a fluorescence microscope (OLYMPUS IX 51) equipped with an OLYMPUS DP 50 camera system and analyzed at 40x magnification.

### 2.8. Statistical Analysis

Three experiments were performed for each analysis. Data are expressed as mean ± SD, and *t*-test was used for the comparison between young and old mice.

## 3. Results and Discussion

### 3.1. Results

**Figure 1:**
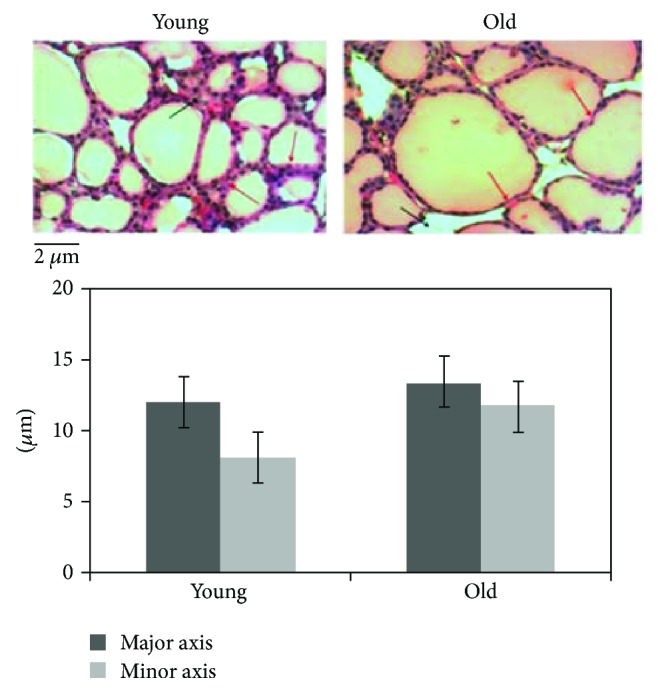
Thyroid morphology in aging. The analysis was performed with hematoxylin-eosin staining. Although some thyroid follicles of old animals appear wider than those of young mouse thyroid, the size of the follicles is not at all homogeneous. The morphometric analysis was performed by using ImageFocus software. Data are reported as mean ± S.D. of three independent experiments performed in duplicate. The results are not statistically significant, as reported in the results. Notably, the structure of thyrocytes and interfollicular spaces appears different in young and old thyroid mice. In particular, thyrocytes are less tall (red arrows) and interfollicular spaces are smaller (black arrows) in old than in young animals. 40x magnification.

**Figure 2:**
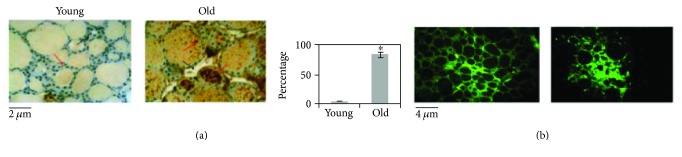
Thyrotropin (TSH) and thyrotropin receptor (TSHR) in the thyroid gland from young and old mice. (a) Immunohistochemical expression of TSH. Red arrows indicate the staining in thyrocytes, and black arrows indicate the staining in interfollicular spaces. In the thyroid of young mice, a low positivity is evident in thyrocytes and it is absent in interfollicular spaces. Strong staining is seen in the majority of follicles either in thyrocytes or in colloid of old mice. The positivity of staining was measured as the percentage of total area (follicles and interfollicular spaces). Data represent the mean ± S.D. of three independent experiments performed in duplicate. Significance, ^∗^*P* < 0.001, versus young mice. 40x magnification. (b) Fluorescence immunostaining of thyrotropin TSHR. The intensity of florescence is higher in old animal than in young animals. Moreover, the fluorescence has a disordered localization in the thyrocytes, within the follicles, and in the interfollicular spaces as shown by arrows. 20x magnification.

**Figure 3:**
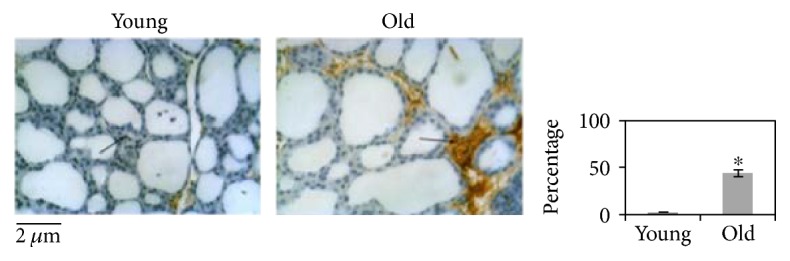
Thyroglobulin in the thyroid gland from young and old mice. Immunohistochemical expression of thyroglobulin. Black arrows indicate staining in interfollicular spaces. In the thyroid of young mice, a low positivity is evident in interfollicular spaces. Strong staining is seen in the interfollicular spaces of old animals. The positivity of staining was measured as the percentage of total area (follicles and interfollicular spaces). Data represent the mean ± S.D. of three independent experiments performed in duplicate. Significance, ^∗^*P* < 0.001, versus young sample. 40x magnification.

**Figure 4:**
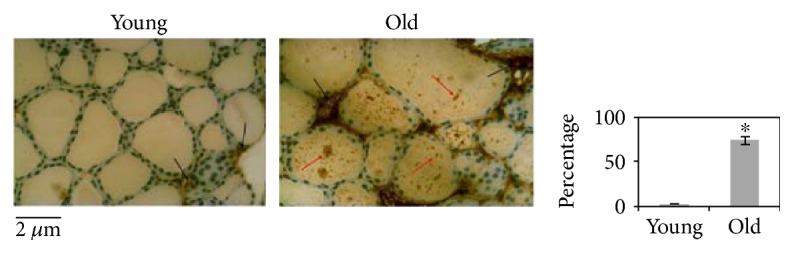
Immunohistochemical expression of galectin-3. Strong staining is seen in the majority of follicles and in interfollicular spaces of old mice, as shown by arrows. The positivity of staining was measured as the percentage of total area (follicles and interfollicular spaces). Data represent the mean ± S.D. of three independent experiments performed in duplicate. 40x magnification. Significance, ^∗^*P* < 0.05, versus young sample.

**Figure 5:**
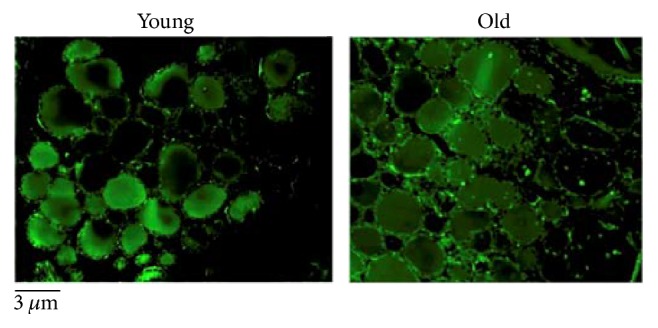
Fluorescence immunostaining of neutral sphingomyelinase (nSMase), by using anti-nSMase antibody in thyroid tissues of young and old mice. The high level of fluorescence is found in the thyrocytes and colloid of young animals. With aging, the fluorescence localization increases in thyrocytes and reduces in colloid. 20x magnification.

In order to study the effect of aging on the thyroid gland, we have first investigated the changes of the microstructure. Morphometric analysis, performed on the thyroid of young and old mice subjected to hematoxylin-eosin staining, shows differences in length of the major axis and of the minor axis of follicles. Since the size of the follicles is not at all homogeneous, the results are not statistically significant (Figure 1). Changes in thyrocyte height and in interfollicular space amplitude are evident. In particular, thyrocytes are less tall and consequently the thyroid epithelium versus colloid volumetric ratio is reduced. Interfollicular cells are poorer in old than in young animals (Figure 1). Then, we have tested the possibility that molecules involved in thyroid function might be associated with structural changes.  Figure 2(a) shows a high content of TSH in old animals accompanied by its abnormal localization in colloid. We next asked whether increasing TSH content in old animals might be associated with modifications of TSHR. To address this question, the immunofluorescence analysis was performed by using specific antibodies against TSHR. Our results demonstrated that the aging induces an increase of TSHR with disordered localization in the thyrocytes, within the follicles, and in the interfollicular spaces (Figure 2(b)). In Figure 3, a strong labeling of thyroglobulin in an interfollicular space is evident in old animals. Since the Gal-3 has been shown to change in the pathological conditions [18], we wondered if this also occurs in aging. To this end, we applied the immunohistochemistry analysis to measure the content and distribution of Gal-3 in the thyroid tissue. We found that the level of Gal-3 is increased in old animals and especially it is abundant in colloid (Figure 4). Following the above finding, we investigated whether nSMase was also changed during the aging process. As shown in Figure 5, nSMase is present in thyrocytes and accumulates in the colloid of young animals; in aging, the nSMase fluorescence is weaker in colloid and increases in thyrocytes.

### 3.2. Discussion

In the human thyroid gland, there is an age-dependent variation of follicular size and expression of iodine transporters [19]. Sorrenti et al. have recently reported an increase of nodular thyroid disease in the elderly [20]. Pasqualett and colleagues [21] reviewed the international scientific literature showing an increase of TSH associated with a reset of the hypothalamus-pituitary-thyroid (HPT) axis. A prompt diagnosis and treatment of HPT axis hypofunction are strongly recommended in elderly patients [22]. Interestingly, our study showed an abnormal distribution of TSH and TSHR inside the follicles. TSH is present in great quantity in the colloid. It is possible that the accumulation of TSH is due to a lack of hormone receptor response. We showed that the TSHR is overexpressed in aging but distributed in a totally disordered manner. Moreover, the thyroglobulin fills the interfollicular spaces which have been increased in volume with aging. The possibility that thyroglobulin is released in the interfollicular spaces because it cannot be used for the synthesis of thyroid hormones cannot be excluded. In this way, our findings might support previous results showing the reduction of thyroid hormone in the blood [19–22]. Prevalence of thyroid dysfunction and its impact on cognition in older adults has been described [23].

However, what molecule is involved in the structure/function alteration of thyroid in aging remains yet unclear. In this study, we provided strong evidence that Gal-3, known to be overexpressed in thyroid cancer [10] by leading to the attenuation of apoptosis [18], is upregulated and moves into colloid during aging, an effect similar to that shown in thyroid damage induced by microgravity conditions [11]. Our results suggest that some thyroid changes occurring during aging parallel modifications are associated with the onset of thyroid gland cancer. Importantly, there are instances in which nSMase is known to mediate alterations in different organs in response to aging, via ceramide production [24]. Thus, we studied the localization of nSMase in the thyroid from young and old animals. The changes of nSMase that result from our study could indicate its possible role in thyroid gland disorders induced by aging. The involvement of nSMase in thyroid damage has been widely described. nSMase in thyroid cells is so important that the enzyme was considered a marker for damage induced during space flights [11]. The exact role of Gal-3 and nSMase is not clear, but the expression levels of both molecules are known to directly stimulate thyroid disorders, as it occurs in microgravity [11]. Our data merely suggest that during aging, not only the HPT axis, the follicular structure, and the synthesis of thyroid hormones are altered as reported in the literature, but also the thyroid gland takes damage similar to cancer as well as damage similar to those induced by microgravity and radiation.

## 4. Conclusions

Our data show an increase in the volume of follicles not statistically significant, an increase in the TSH expression that fills the colloid, and in thyroglobulin that diffuses in the interfollicular spaces. Moreover, TSHR also appears upregulated. Notably, Gal-3 and nSMase are upregulated and delocalized in comparison with control mice. Taken together, these data suggest that not only the follicle structure and molecules involved in the synthesis of thyroid hormones change in aging, by supporting previous data [19–22], but also molecules involved in the thyroid damages, such as Gal-3 [11, 18] and nSMase [11–24]. To our knowledge, this is the first study correlating thyroid changes in aging and markers of thyroid damages including cancer. Here, we found higher expression of Gal-3 and a redistribution of nSMase in the thyroid of old animals in comparison with those of young animals. This body of work suggests that Gal-3 and nSMase, by inducing thyroid changes, might be the cause of the increased expression and altered distribution of TSH, thyroglobulin, and TSHR or vice-versa. Future studies will clarify this point.
